# Analysis of the Evolution of Pandemic Influenza A(H1N1) Virus Neuraminidase Reveals Entanglement of Different Phenotypic Characteristics

**DOI:** 10.1128/mBio.00287-21

**Published:** 2021-05-11

**Authors:** Meiling Dai, Wenjuan Du, Carles Martínez-Romero, Tim Leenders, Tom Wennekes, Guus F. Rimmelzwaan, Frank J. M. van Kuppeveld, Ron A. M. Fouchier, Adolfo Garcia-Sastre, Erik de Vries, Cornelis A. M. de Haan

**Affiliations:** a Virology Section, Division of Infectious Diseases & Immunology, Department of Biomolecular Health Sciences, Faculty of Veterinary Medicine, Utrecht University, Utrecht, the Netherlands; b Department of Microbiology, Icahn School of Medicine at Mount Sinai, New York, New York, USA; c Global Health and Emerging Pathogens Institute, Icahn School of Medicine at Mount Sinai, New York, New York, USA; d Department Chemical Biology and Drug Discovery, Utrecht Institute for Pharmaceutical Sciences and Bijvoet Center for Biomolecular Research, Utrecht University, Utrecht, The Netherlands; e Research Center for Emerging Infections and Zoonoses, University of Veterinary Medicine, Hannover, Germany; f Department of Viroscience, Erasmus Medical Center, Rotterdam, the Netherlands; g Department of Medicine, Division of Infectious Diseases, Icahn School of Medicine at Mount Sinai, New York, New York, USA; h The Tisch Cancer Institute, Icahn School of Medicine at Mount Sinai, New York, New York, USA; The Peter Doherty Institute for Infection and Immunity

**Keywords:** antigenicity, enzymatic activity, influenza A virus, neuraminidase

## Abstract

The influenza A virus (IAV) neuraminidase (NA) is essential for virion release from cells and decoy receptors and an important target of antiviral drugs and antibodies. Adaptation to a new host sialome and escape from the host immune system are forces driving the selection of mutations in the NA gene. Phylogenetic analysis shows that until 2015, 16 amino acid substitutions in NA became fixed in the virus population after introduction in the human population of the pandemic IAV H1N1 (H1N1pdm09) in 2009. The accumulative effect of these substitutions, in the order in which they appeared, was analyzed using recombinant proteins and viruses in combination with different functional assays. The results indicate that NA activity did not evolve to a single optimum but rather fluctuated within a certain bandwidth. Furthermore, antigenic and enzymatic properties of NA were intertwined, with several residues affecting multiple properties. For example, the substitution K432E in the second sialic acid binding site, next to the catalytic site, was shown to affect catalytic activity, substrate specificity, and the pH optimum for maximum activity. This substitution also altered antigenicity of NA, which may explain its selection. We propose that the entanglement of NA phenotypes may be an important determining factor in the evolution of NA.

## INTRODUCTION

Influenza viruses (IAVs) are human respiratory pathogens that cause seasonal epidemics and occasional pandemics. Pandemics can occur when animal IAVs cross the host species barrier and are efficiently spread between humans. In subsequent years, these viruses give rise to seasonal epidemics with significant disease and mortality. The only IAV pandemic of the 21st century so far started in April 2009 and was caused by a novel swine origin H1N1 IAV (H1N1pdm09) (1). This first influenza pandemic in the genomics era allows us to follow the evolution of a pandemic IAV spreading in the human population in detail. This evolution may entail selection of substitutions in viral proteins driven by adaptation to the new host and selective pressure by the host immune system.

IAV particles contain two glycoproteins, the hemagglutinin (HA), which is a receptor-binding and fusion protein, and the neuraminidase (NA) protein, which has receptor-destroying activity. Both proteins are important determinants of host tropism, pathogenesis, and transmission and are prime targets of the host immune system. HAs of avian viruses preferentially bind to sialic acids (SIAs) linked to the proximal galactose by an α2,3 bond (avian-type receptor) (2, 3). Human IAVs, including the H1N1pdm09 virus, preferentially bind to α2,6-linked SIAs (human-type receptor), which are abundant on epithelial cells of the human upper respiratory tract (4, 5). The IAV NA protein cleaves SIAs from glycoproteins and glycolipids. The NA protein thereby facilitates release of progeny virions from infected cells and decoy receptors (e.g., in mucus) and prevents virus self-aggregation (6). Several studies indicate that a functional balance between HA binding and NA cleavage is important for maintaining optimal virus replication as well as transmission across different host species (7–13, 60). Knowledge of the molecular details of the HA-NA balance, and particularly the role of NA therein, is, however, still largely lacking.

The NA protein is a type II membrane-anchored glycoprotein that forms homotetramers. The NA ectodomain contains a thin stalk and a globular head domain containing a six-bladed beta-propeller structure. SIA cleavage is mediated by the active site located in the NA head domain (14). The NA active site is composed of several highly conserved catalytic residues that directly contact the SIA substrate as well as structural residues that stabilize catalytic residues in place (15, 16). The NA protein of H1N1pdm09 preferentially cleaves α2,3- over α2,6-linked SIAs (9, 12, 17, 18) with an optimal pH range of 5.5 to 6.5 (16, 19). Ca^2+^ is required for NA catalytic activity and thermostability (16), and three Ca^2+^-binding sites have been identified for the NA protein of H1N1pdm09 (20). NA catalytic activity for multivalent substrates is enhanced by the presence of a second SIA binding site (2SBS) (12, 13, 21, 22), a shallow pocket composed of three surface loops located adjacent to the active site (23; for a review, see reference 24). The 2SBS of N1, N2, and N9 proteins preferably binds α2,3-linked sialosides (12, 13, 22). Five of the six SIA contact residues in the 2SBS identified by structural analysis of the N9 protein (23) are highly conserved among the NAs of avian IAVs, but this conservation is lost in the N1 and N2 proteins of human seasonal IAVs (12, 13, 21, 23, 25), including the H1N1pdm09 virus. In agreement herewith, the N1 proteins of H1N1pdm09 and seasonal human H1N1 IAVs display severely reduced binding to α2,3-sialyllactose compared to their avian counterparts, as determined by saturation-transfer difference nuclear magnetic resonance (STD-NMR) (26) as well as reduced enzymatic activity against multivalent substrates (12).

The NA protein is an important target for antiviral drugs and antibodies contributing to protection against influenza (27–33). The phenotypic evolution of NA of H1N1pdm09 in the human population is not well studied, even though understanding evolution of NA-directed immunity may contribute to the design of better IAV vaccines (34). Possibly, alterations of NA activity are linked to changes in antigenicity, as has been observed for HA (35, 36). Analysis of antigenic drift of H1N1pdm09 N1 suggests a role for substitutions at position 386, 390, and 432 in antigenicity changes (31, 37). These residues have been shown to be important for the binding of monoclonal antibodies (31, 38–41). However, the effect of most of these substitutions on NA enzymatic activity is not known, even though this probably plays an important role in their selection. In the current study, we studied the evolution of H1N1pdm09 N1 activity and antigenicity and analyzed to what extent different phenotypic properties of NA are intertwined. Therefore, we performed phylogenetic analysis of H1N1pdm09 NA sequences deposited between 2009 and 2015 to identify amino acid substitutions that were conserved over time. Using different functional assays, we analyzed the cumulative effect of those substitutions in chronological order. Our results indicate that NA functionality fluctuates within a certain bandwidth and does not evolve to attain maximum activity. Furthermore, several substitutions were shown to affect several phenotypic characteristics of NA, including enzymatic activity and antigenicity. Of note, we identified an important role for the residue at position 432 in the 2SBS adjacent to the catalytic site, as substitution of this residue was shown to affect multiple aspects of NA activity as well as antigenicity. The entanglement of NA phenotypes is proposed to be an important determining factor in the evolution of this protein.

## RESULTS

### Evolution and expression of NA of H1N1pdm09 from 2009 until 2015.

**FIG 1:**
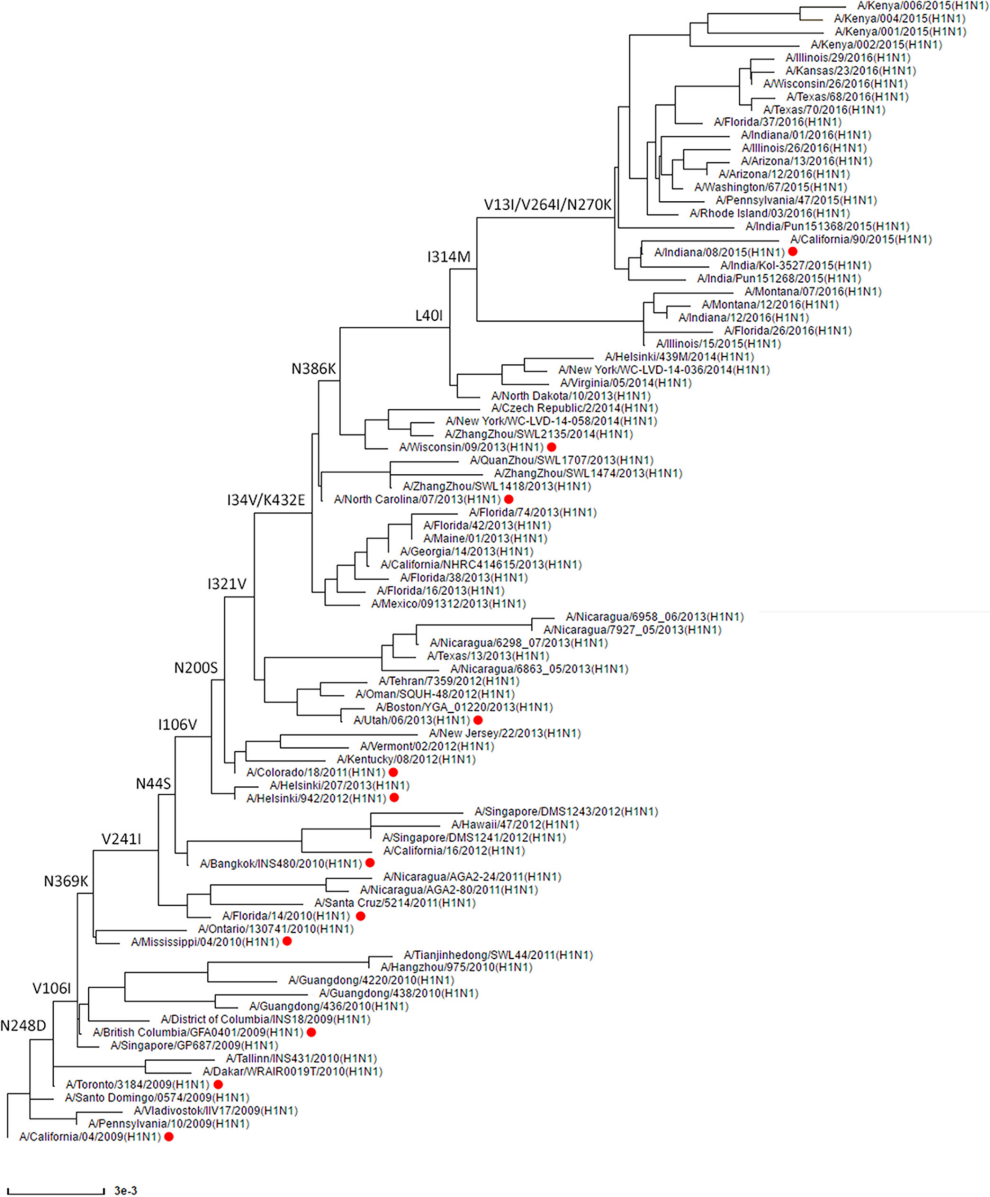
Phylogenetic analysis of H1N1pdm09 N1 proteins from 2009 until 2015. All full-length and unique N1 protein sequences of H1N1pdm09 were downloaded from the NCBI database and used to construct an N1 gene guide tree, which was used to select N1 sequences representing all main branches of the tree. The selected N1 genes were used to construct a summary tree with a topology similar to that of the guide tree. The N1 tree is rooted by the A/California/04/2009 isolate. Substitutions specified on the tree backbone indicate specific protein substitutions that became fixed in the virus population. Red dots indicate virus isolates whose NA proteins contain only the indicated substitutions and were expressed in this study. An alignment of these proteins is shown in Fig. S1, while the substitutions are also listed in Table 1.

**FIG 2:**
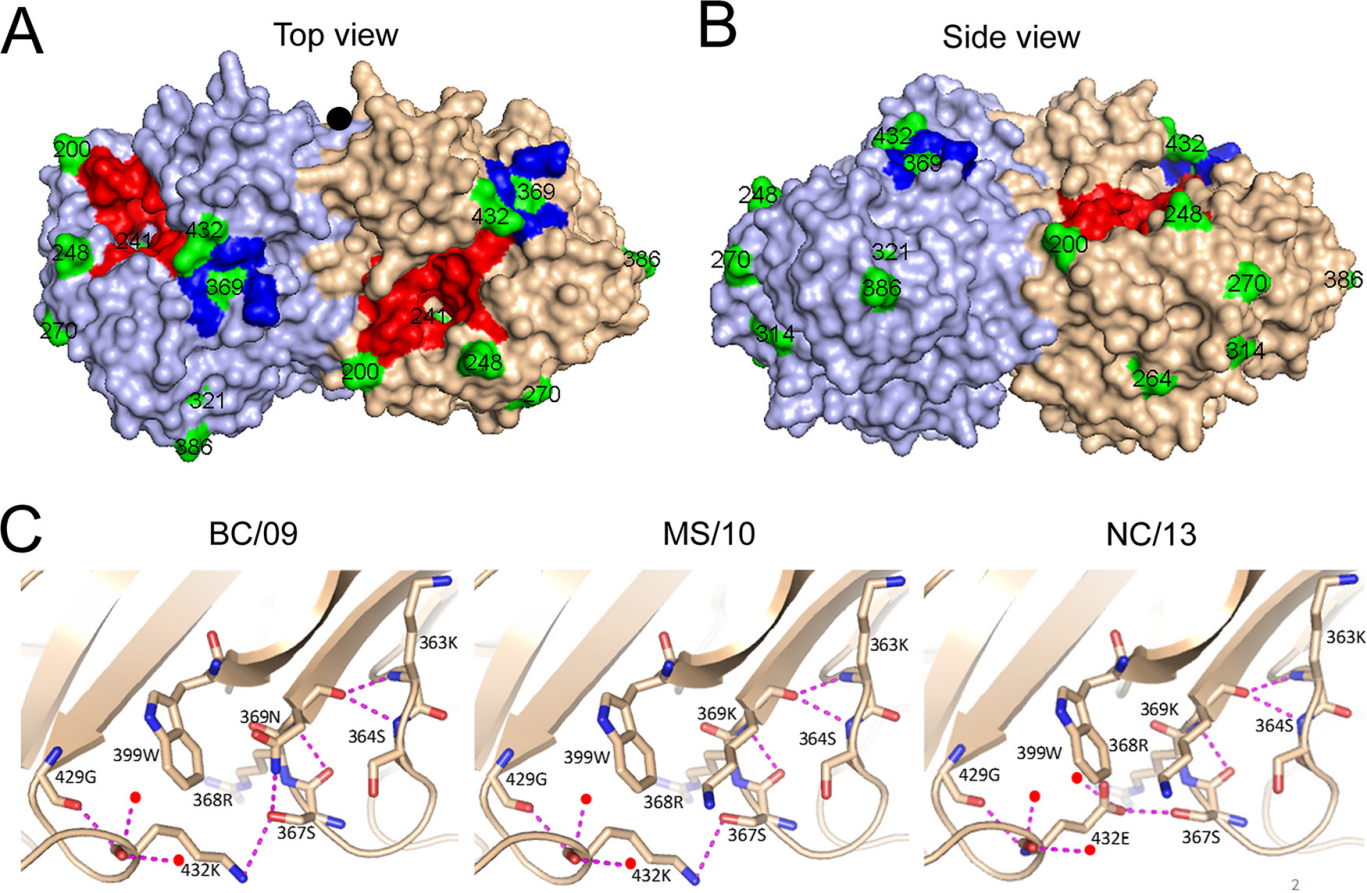
Structural analysis of the N1 proteins. Top (A) and side (B) views of the structure of the NA protein from A/California/04/2009 (PDB 3NSS) generated with PyMOL software (20). The NA active site and the second SIA-binding site are colored red and blue. The amino acids in the NA head domain whose substitutions (Fig. 1) became fixed in the population are colored green, and their numbering is indicated. V241I is located underneath the active site but does not belong to the catalytic or framework residues. K432E and N369K are located in the 2SBS. The residue at position 106 is not visible in this surface representation. The black circle indicates the 4-fold symmetr*y* axis. The substitutions are also indicated in the alignment shown in Fig. S1 and in Table 1. (C) Close-up views of the structures of the indicated NAs in cartoon representation, with the indicated amino acids shown as sticks (oxygen in red, nitrogen in blue). Water molecules are shown as red spheres and hydrogen bonds as dashed lines.

To get more insight into the phenotypic evolution of NA of H1N1pdm09 virus, we first reconstructed the evolutionary path of NA starting with the emergence of the H1N1pdm09 virus in April 2009 by generating an NA phylogenetic tree (Fig. 1). Analysis of the different NA gene sequences revealed that 16 amino acid substitutions (N248D, V106I, N369K, V241I, N44S, I106V, N200S, I321V, I34V/K432E, N386K, L40I, I314M, and V13I/V264I/N270K) became fixed in the virus population (Fig. 1 and Table 1). For some substitutions (I34V/K432E and V13I/V264I/N270K), the order in which they were acquired could not be resolved. The location of these substitutions in the NA, excluding the substitutions located in the transmembrane domain or the stalk region (V13I, I34V, L40I, and N44S), the structure of which is not resolved, is shown in Fig. 2. Although some substitutions are located relatively close to the active site (N200S, N248D, V241I, and K432E), none of them involves catalytic or framework residues (15, 16) (Fig. 2). The substitutions N369K and K432E are located in the 2SBS at positions known to interact with SIA according to an N9 crystal structure (23). The other residues, except the residue at position 106, are scattered across the surface of the NA head domain. Of note, N386K and N44S disrupt and generate a potential N-glycosylation site, respectively. Residue 386 is also part of a Ca^2+^-binding site, while residue 106 is located close to the subunit interfaces and to the Ca^2+^-binding site located at the 4-fold axis (19, 42, 43).

**TABLE 1:** NA proteins analyzed

Abbreviation	Isolate^*a*^	Substitution(s) introduced^*b*^	Accumulative substitutions^*c*^
CA/09	A/California/04/2009		
TO/09	A/Toronto/3184/2009	N248D	N248D
BC/09	A/British Columbia/GFA0401/2009	V106I	N248D+V106I
MS/10	A/Mississippi/04/2010	N369K	N248D+V106I+N369K
FL/10	A/Florida/14/2010	V241I	N248D+V106I+N369K+V241I
BK/10	A/Bangkok/INS480/2010	N44S	N248D+V106I+N369K+N44S+V241I
HS/12	A/Helsinki/942/2012	I106V	N248D+V106I+N369K+N44S+V241I+I106V
CO/11	A/Colorado/18/2011	N200S	N248D+V106I+N369K+N44S+V241I+I106V+N200S
UT/13	A/Utah/06/2013	I321V	N248D+V106I+N369K+N44S+V241I+I106V+N200S+I321V
NC/13	A/North Carolina/07/2013	K432E	N248D+V106I+N369K+N44S+V241I+I106V+N200S+I321V+K432E
WI/13	A/Wisconsin/09/2013	N386K	N248D+V106I+N369K+N44S+V241I+I106V+N200S+I321V+K432E+N386K
WI/13-314		I314M	N248D+V106I+N369K+V241I+N44S+I106V+N200S+I321V+K432E+N386K+I314M
IN/15	A/Indiana/08/2015	V264I+N270K	N248D+V106I+N369K+V241I+N44S+I106V+N200S+I321V+K432E+N386K+I314M+V264I+N270K

a Virus isolates carrying NA proteins identical to those analyzed in this study are listed. These isolates correspond to the isolates indicated with the red dots in Fig. 1.

b The substitution(s) introduced relative to the precursor virus is given.

c Accumulative substitutions are given. Please note that viruses starting with HS/12 contain V at position 106, similar to CA/09 and TO/09.

10.1128/mBio.00287-21.1 FIG S1 Alignment of NA of H1N1pdm09. Alignment of the N1 proteins analyzed in this study. Differences between the different proteins are highlighted in green. Active-site residues (including both residues that have direct interaction with the substrate and framework residues that stabilize the catalytic site) are indicated in red (G. M. Air, Influenza Other Respir Viruses 6:245–256, 2012, https://doi.org/10.1111/j.1750-2659.2011.00304.x). Residues that form the Ca^2+^ binding sites (site 1, positions 111 and 113; site 2, positions 293, 297, 324, 345, and 347; site 3, positions 376, 379, 384, and 386) are indicated in yellow (X. Xu, X. Zhu, R. A. Dwek, J. Stevens, and J. A. Wilson, J Virol 82:10493–10501, 2008, https://doi.org/10.1128/JVI.00959-08). Residues corresponding to residues in the N9 protein that have direct interaction with SIA in the second SIA-binding site are in highlighted in blue (J. N. Varghese, P. M. Colman, A. van Donkelaar, T. J. Blick, et al., Proc Natl Acad Sci U S A 94:11808–11812, 1997, https://doi.org/10.1073/pnas.94.22.11808). The transmembrane domain is indicated in grey, while N-glycosylation sites are indicated by “Glyc.” The NA starting residue of the recombinant soluble NA proteins is indicated by the blue rectangle (N42). Download FIG S1, TIF file, 0.9 MB. Copyright © 2021 Dai et al. 2021 Dai et al. https://creativecommons.org/licenses/by/4.0/ This content is distributed under the terms of the Creative Commons Attribution 4.0 International license.

To evaluate the phenotypic changes in NA functionality associated with these substitutions, they were introduced into recombinant soluble tetrameric versions of H1N1pdm09 NA (12) (starting at position 42) in accumulative order according to the phylogenetic analysis (Table 1). Viruses carrying these NA proteins are indicated in Fig. 1, while an NA alignment is shown in Fig. S1 in the supplemental material. The NA proteins are abbreviated with two letters that refer to the location and two digits that refer to the year of isolation. In addition, we made a recombinant NA protein containing the I314M substitution in the background of the WI/13 NA protein. As this substitution is found in combination with other (nonfixed) substitutions, this NA protein is referred to as WI/13-314.

### Cleavage of monovalent substrates.

**FIG 3:**
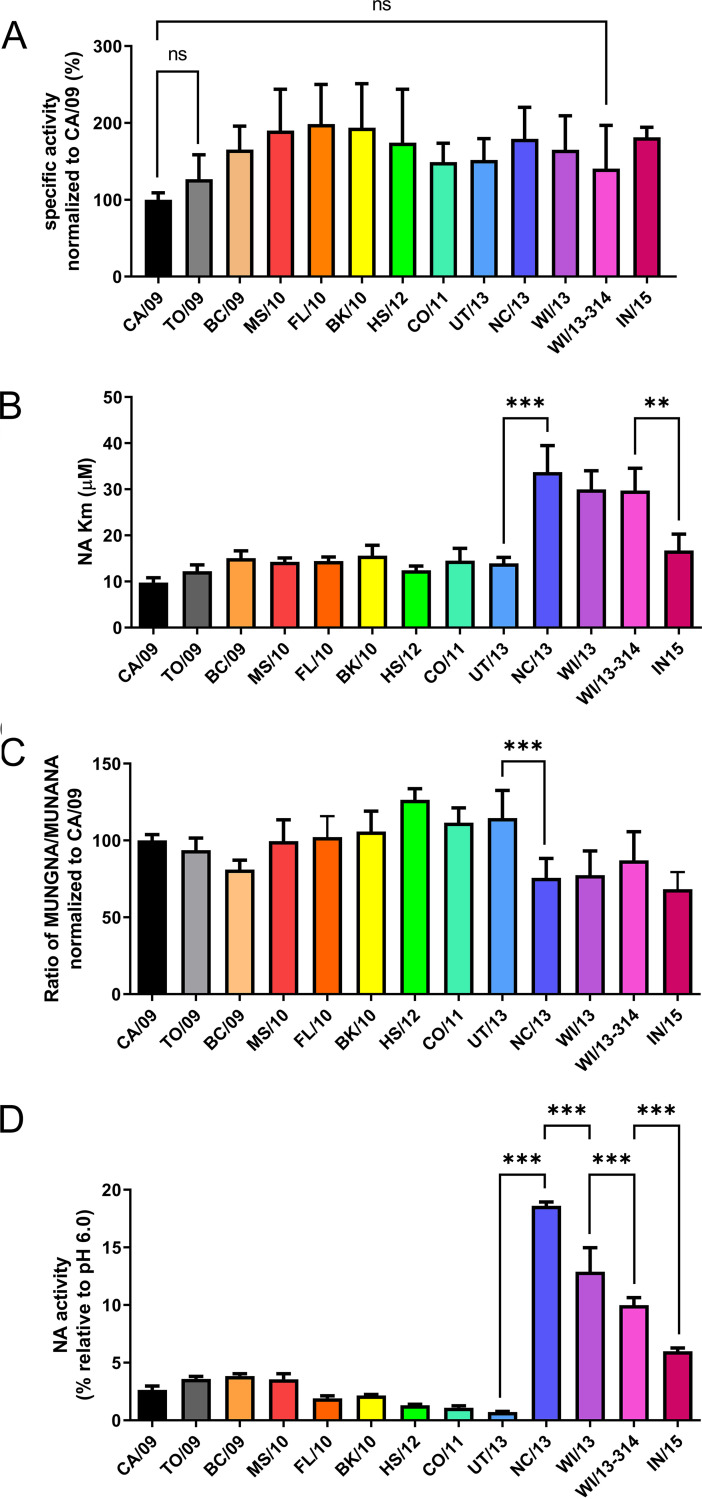
Activity of H1N1pdm09 NA proteins using monovalent substrates. (A) Specific activity of indicated NA proteins using the substrate MUNANA is graphed normalized to the specific activity of CA/09 NA. (B) *K_m_* values of the indicated NA proteins for MUNANA. None of the NA proteins significantly differ in their specific activity relative to their precursor. All NA proteins do differ, however, from CA/09, with the exceptions of TO/09 and WI13-314 (indicated by “ns” [nonsignificant]). (C) Ratio of the specific activity of the indicated NA proteins using the Neu5Gc-containing MUNGNA and the Neu5Ac-containing MUNANA (MUNGNA/MUNANA) normalized to that of CA/09 NA. (D) The activity of the different NA proteins was determined at pH 4.6 using the MUNANA assay and graphed relative to the activity at pH 6.0. The graphs the means from 2 to 6 independent experiments performed in triplicate. Error bars indicate standard deviations. (B to D) For each NA protein, significant differences relative to its precursor NA are indicated (*, *P* < 0.05; **, *P* < 0.01; ***, *P* < 0.001).

While humans synthesize only *N*-acetylneuraminic acid (Neu5Ac)-containing sialoglycan receptors, swine also express *N*-glycolylneuraminic acid (Neu5Gc) (44). As substitutions in NA may result from adaptation of the H1N1pdm09 virus to the human SIA receptor repertoire, the enzymatic properties of NA were analyzed using monovalent substrates that represent either Neu5Ac (MUNANA) or Neu5Gc (MUNGNA). The specific activity (activity/amount of protein) of the NAs for Neu5Ac-containing substrate gradually increased to a 1.5- to 2-fold-higher level by the cumulative addition of NA substitutions (Fig. 3A). None of the NAs displayed significantly altered specific activity compared to their precursor protein, although all NAs were significantly more active than CA/09 except TO/09 and WI/13-314. After introduction of the K432E substitution (NC/13), an increased *K_m_* value (i.e., decreased substrate affinity) was observed using the MUNANA substrate (Fig. 3B). This higher *K_m_* value was maintained in the other proteins containing this substitution, except IN/15. All NA proteins displayed the same ratio of specific activities comparing MUNANA to MUNGNA substrates, with the exception again of the NA proteins containing the K432E substitution, which consistently had a decreased ability to cleave MUNGNA (Fig. 3C). The ability of NA to cleave sialosides at low pH during virus entry has been shown to enhance virus replication *in vitro* (45, 46) and was suggested to contribute to the spread of pandemic viruses (19, 46). Hence, we analyzed the enzymatic activity of the NA proteins at pH 4.6 compared to the activity at the routinely used pH 6.0 using the MUNANA assay. Preliminary analyses indicated that the recombinant proteins display similar catalytic activities at pH 5.0 and 6.0. NA activity at pH 4.6 was relatively low for the proteins with K432 (CA/09 up to and including UT/13) and high for the proteins containing E432 (NC/13, WI/13, WI/13-314, and IN/15) (Fig. 3D). Thus, the K432E substitution (introduced in NC/13) has a positive effect on catalytic activity at low pH. As several substitutions are located at or close to Ca^2+^-binding sites known to be important for thermal stability (43, 47–49), we analyzed the thermostability of NA activity by incubating NA at 50°C for 5 min in the absence or presence of EDTA, after which the activity was measured using the MUNANA assay (Fig. S2). The results indicate that residues 106 and 369 are determinants of Ca^2+^-dependent thermostability. Substitutions that affected Ca^2+^-dependent thermostability did not affect the relative activity of the NA at low pH and *vice versa*.

10.1128/mBio.00287-21.2 FIG S2 Ca^2+^-dependent thermostability of H1N1pdm09 NAs. Ca^2+^-dependent thermostability was analyzed by determining the NA activity using the MUNANA assay after heating NA samples for 5 min at 50°C in buffer lacking Ca^2+^ and containing EDTA. Values are graphed relative to unheated controls. Representative results of experiments performed in triplicate are shown. For each NA protein, significant differences relative to its precursor protein are indicated (*, *P* < 0.05; **, *P* < 0.01; ***, *P* < 0.001). Download FIG S2, TIF file, 1.3 MB. Copyright © 2021 Dai et al. 2021 Dai et al. https://creativecommons.org/licenses/by/4.0/ This content is distributed under the terms of the Creative Commons Attribution 4.0 International license.

### Cleavage of multivalent substrates.

**FIG 4:**
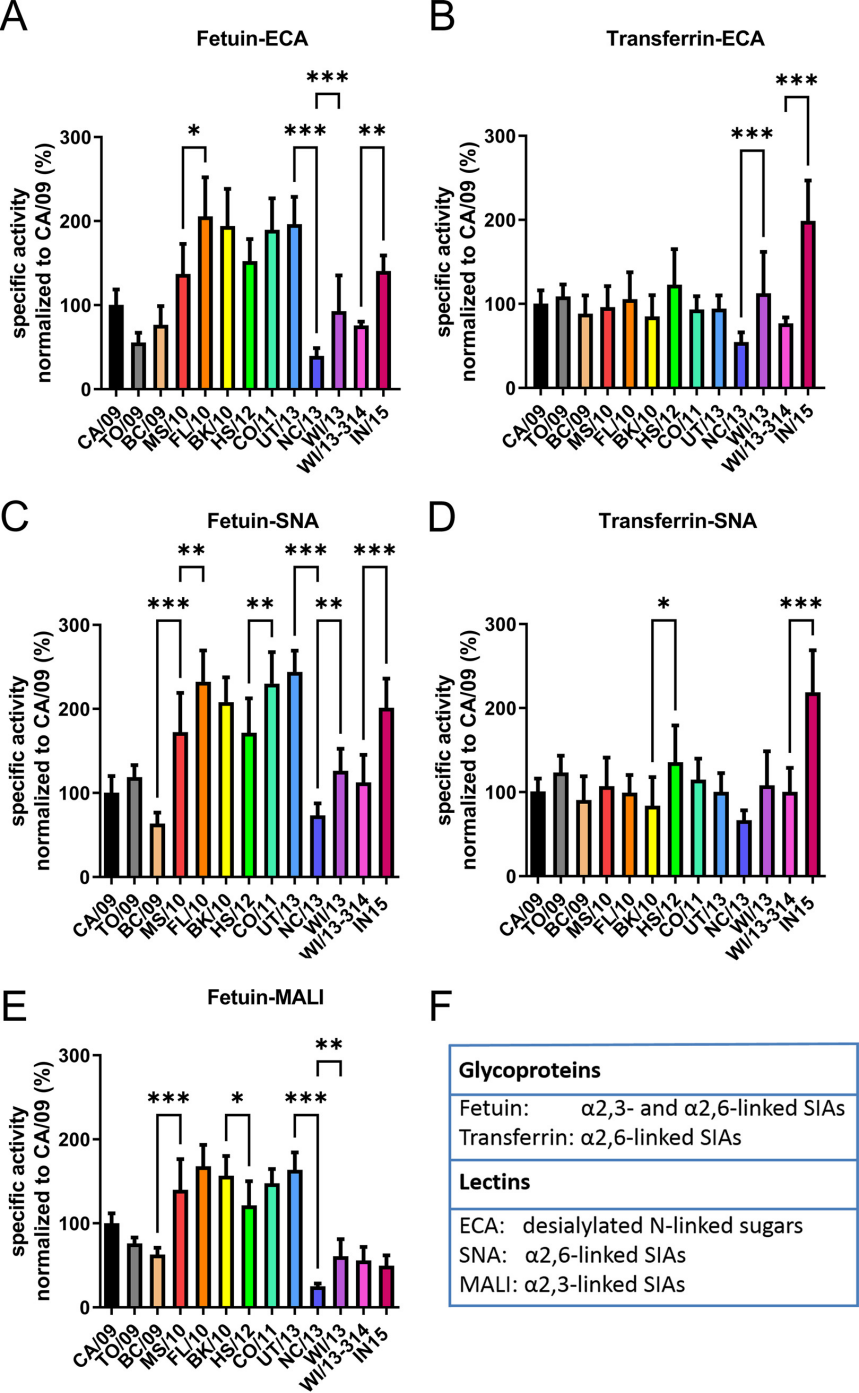
Specific activities of H1N1pdm09 NA proteins for multivalent substrates. Specific activities of the indicated H1N1pdm09 NA proteins were determined by ELLA using different glycoprotein-lectin combinations: (A) fetuin-ECA, (B) transferrin-ECA, (C) fetuin-SNA, (D) transferrin-SNA, and (E) fetuin-MALI. Results were normalized to the activity of NA CA/09. Means of at least three independent experiments performed in duplicate/triplicate are shown. Standard deviations are indicated. For each NA protein, significant differences relative to its precursor protein are indicated (*, *P* < 0.05; **, *P* < 0.01; ***, *P* < 0.001). (F) Information about the fetuin, transferrin, and the binding preference of lectins used in this study.

The enzymatic activity of the different NAs was analyzed with the glycoproteins fetuin and transferrin as substrates using enzyme-linked lectin assays (ELLAs) as described previously (12, 22). The multivalent presentation of glycans on fetuin and transferrin resembles the *in vivo* presentation of SIAs on cell surface-attached glycoproteins, in contrast to the soluble monovalent substrates used above. Fetuin contains mono-, bi-, and triantennary glycans with α2,3- and α2,6-linked SIAs (50). Transferrin contains two biantennary N-linked glycan chains with only α2,6-linked SIAs (51, 52). Cleavage of SIAs from fetuin and transferrin by serially diluted NA was quantified by lectin binding in two ways, using either increase of Erythrina crista-galli lectin (ECA) binding or decrease of Maackia amurensis lectin I (MALI) or Sambucus nigra lectin (SNA) binding as a readout. ECA specifically recognizes glycans containing terminal Galβ1-4GlcNAc, which become exposed upon desialylation of N-linked sugars (53). MALI and SNA specifically bind α2,3- and α2,6-linked SIAs, respectively (54, 55). Despite some differences between different fetuin-lectin combinations (Fig. 4), the results consistently show a positive effect of the N369K substitution (introduced in MS10) and a large negative effect of the K432E substitution (introduced in NC/13) on the cleavage of α2,3- and α2,6-linked SIA from fetuin. Another consistent effect was the modestly increased specific activity resulting from the N386K substitution (introduced in WI/13). No positive effect of the N369K substitution was observed when transferrin was used as the substrate, while the negative effect of the K432E substitution was much smaller and not significantly different (Fig. 4B and D). In contrast, the positive effect of the N386K substitution was still observed when transferrin was used (Fig. 4B). N386K results in the loss of a putative glycosylation site (Fig. S1). Gel-electrophoretic analysis of NC/13 and WI/13 proteins, differing only at position 386 (Fig. S3), indicated, however, that the N386 residue is not modified by the addition of an *N*-glycan. The substitutions V264I and N270K in IN/15 consistently resulted in increased cleavage of α2,6-linked, but not α2,3-linked, SIAs, which was observed for both fetuin and transferrin.

10.1128/mBio.00287-21.3 FIG S3 N-glycosylation of H1N1pdm09 NAs. (A) Recombinant soluble NC/13 and WI/13 proteins expressed in HEK293S GnTI(−) cells were analyzed by gel electrophoresis followed by Western blotting using an antibody against the Strep tag (StrepMabClassic-HRP [IBA]). NC/13 and WI/13 run at a similar position in the gel before and after PNGase F treatment, which removes *N*-glycans, indicating that the N386 residue is not modified by the addition of an *N*-glycan in NC/13. The position on the gel of the relevant molecular weight markers is shown on the left side of the gels. Download FIG S3, TIF file, 0.2 MB. Copyright © 2021 Dai et al. 2021 Dai et al. https://creativecommons.org/licenses/by/4.0/ This content is distributed under the terms of the Creative Commons Attribution 4.0 International license.

Previously, we showed that increased cleavage of multivalent substrates correlated with increased binding of NA to glycans via the 2SBS to α2,3-linked SIAs (12, 13, 22), whereas activity on monovalent substrates was not affected. Here, the substitutions at positions 369 and 432, located in the 2SBS of the N1 protein, affected the specific activity only when the multivalent substrate fetuin (Fig. 4A) was used, not when soluble monovalent MUNANA (Fig. 3A) or transferrin, which contains only α2,6-linked SIAs (Fig. 4B), was used (note that the 2SBS binds much better to α2,3-linked SIAs). Therefore, we also analyzed the hemagglutinating ability of membrane vesicles containing UT/13 (with K432) and NC/13 (with E432) NA as described previously (13) (Fig. S4). Both proteins did not display hemagglutinating activity, in contrast to an avian N1, indicating a very low avidity for the interaction of NA of H1N1pdm09 with sialosides via the 2SBS regardless of the identity of the residue at position 432. Still, considering the effect of substitutions in the 2SBS when using fetuin (α2,3-linked SIAs) in the ELLA (Fig. 4A), we conclude that a very weak interaction of α2,3-linked SIAs with 2SBS contributes to NA activity on multivalent substrates.

10.1128/mBio.00287-21.4 FIG S4 Hemagglutination assay of NA membrane vesicles. Hemagglutination assays were performed with membrane vesicles containing similar amounts of NA activity of either the indicated H1N1pdm09 NAs (UT/13 and NC/13) or NAs (N1_Hunan_ and N1_432E_) from H5N1 viruses (Du et al., submitted) as described previously (13). The NA proteins from H5N1 viruses, which serve as positive and negative controls, have a higher catalytic activity than NA from CA/09 (12), resulting in larger protein amounts for the H1N1pdm09 NAs than the NAs from H5N1 viruses. Twofold serial dilutions of the vesicles were incubated with equal volumes of 0.5% human erythrocytes at 4°C for 2 h in the presence of OC. Red dots at the bottom of the wells indicate the absence of hemagglutination. Results of a representative experiment (out of three performed) are shown. Download FIG S4, TIF file, 1.6 MB. Copyright © 2021 Dai et al. 2021 Dai et al. https://creativecommons.org/licenses/by/4.0/ This content is distributed under the terms of the Creative Commons Attribution 4.0 International license.

### Effect of N1 substitutions on virus replication and NA activity of virions.

**FIG 5:**
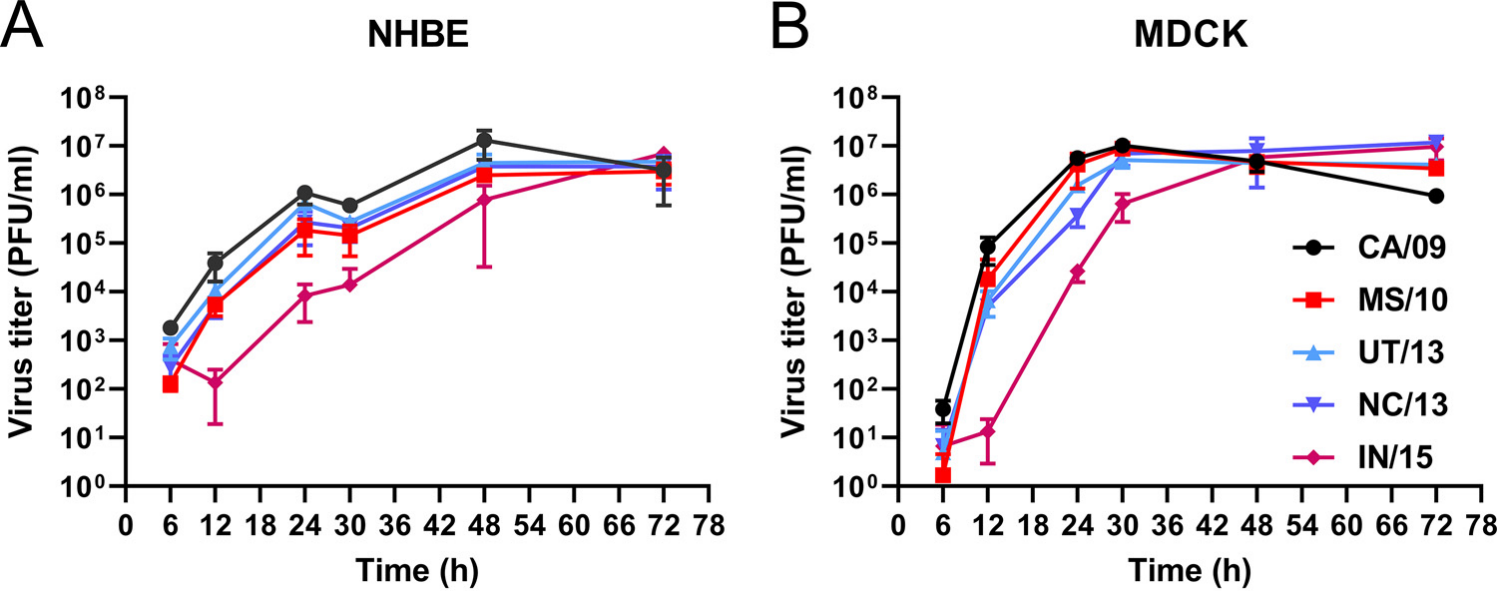
Replication kinetics of recombinant H1N1N viruses. NHBE (A) and MDCK (B) cells were infected with recombinant H1N1 viruses carrying different NA proteins at multiplicities of infection (MOI) of 0.1 and 0.001, respectively. Virus in the cell culture supernatants at the indicated times postinfection was harvested and titrated on MDCK cells, and the titers were expressed as PFU per milliliter.

To investigate the effect of the substitutions in NA in the context of virus particle, five recombinant viruses were generated in the background of A/California/04/2009 pdmH1N1. These viruses, which contain either CA/09, MS/10, UT/13, NC/13, or IN/15 NA, differ only in their NA genome segments. The replication of these viruses in human bronchial epithelial cells (NHBE) and Madin-Darby canine kidney (MDCK) cells was assessed (Fig. 5). Three viruses produced virus titers similar to those of CA/09 virus, whereas IN/15 virus titers were somewhat lower at early time points.

**FIG 6:**
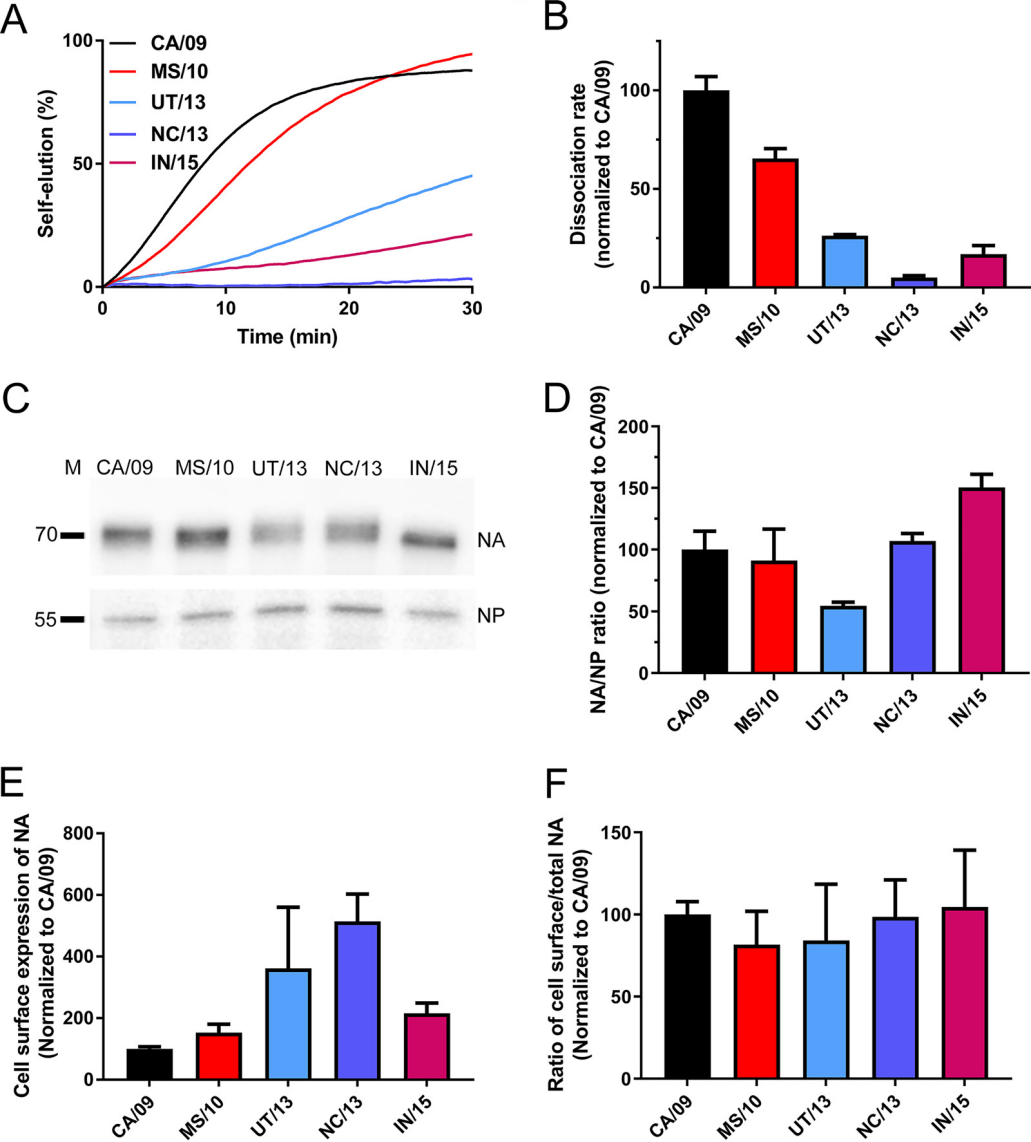
NA enzymatic activity of recombinant viruses. (A) Recombinant viruses were allowed to bind to similar levels to sensors containing 6′LAMP1 in the presence of OC. After removal of OC by short washes, virion self-elution in the absence of OC was monitored. Dissociation of virus particles was normalized to the virus association levels in the presence of OC. Results of a representative experiment out of three is shown. (B) The maximum slope of the virus dissociation curve (shown in panel A) was calculated and normalized to that of the CA/09 virus. (C) Western blot analysis of NA and NP in virus preparations pelleted through a 20% sucrose cushion using monoclonal antibodies against NA and NP. The position of molecular mass markers is indicated on the left side of the gel (D) The intensity of the NA and NP protein bands was quantified using the Odyssey gel imaging system (Li-Cor), and the mean ratio (of two independent experiments) of NA/NP normalized to that of CA/09 is graphed. (E) Enzymatic activity of cell surface-expressed NAs using the MUNANA assay. (F) Ratio of cell surface-expressed NA to total cell-associated NA was determined by using the MUNANA assay. All results are means from three independent experiments. Standard deviations are indicated.

Using a recently established biolayer interferometry (BLI)-based kinetic assay (13, 56), the contribution of the different NAs to the HA-NA balance of virus particles was determined by analyzing NA-driven virion self-elution. For viruses carrying the same HA, kinetics of self-elution are determined by NA enzymatic activity of virions. Sensors coated with α2,6-sialylated lysosome-associated membrane glycoprotein 1 (6′LAMP1) were loaded to equal levels with the different recombinant viruses in the presence of the NA inhibitor oseltamivir-carboxylate (OC), after which NA-driven virion self-elution in the absence of OC was monitored (Fig. 6A and B). Viruses carrying CA/09 NA displayed the fastest self-elution. Substitutions in NA reduced virion self-elution, which was slowest for NC/13 virus. Additional substitutions in IN/15 increased virion-self elution again (Fig. 6A and B). Next, we analyzed the incorporation of the different NAs into virus particles, by performing Western blot analysis using specific antibodies against NA and NP (Fig. 6C and D). With the exception of UT/13 NA, the incorporation of NA into virions was not negatively affected compared to CA/09 NA.

To check whether the reduced incorporation of UT/13 NA was due to reduced intracellular NA trafficking and cell surface expression, the enzymatic activity of cell surface and intracellular full-length NA was quantified by using the MUNANA assay. The results indicate that the different NAs appear positively affected in their cell surface expression compared to CA/09 (Fig. 6E). The different NAs displayed a similar ratio of cell surface to intracellular enzymatic activity, indicating that intracellular trafficking was not negatively affected (Fig. 6F). Apparently, reduced incorporation of UT/13 NA compared to CA/09 NA does not result from reduced intracellular trafficking and cell surface expression. We conclude that the reduced self-elution of the UT/13 virus correlates with reduced incorporation of UT/13 NA into virions. The reduced self-elution of the NC/13 virus correlates with the NC/13 NA having a low catalytic activity against multivalent substrates (Fig. 4).

### Antigenicity of N1 proteins.

**FIG 7:**
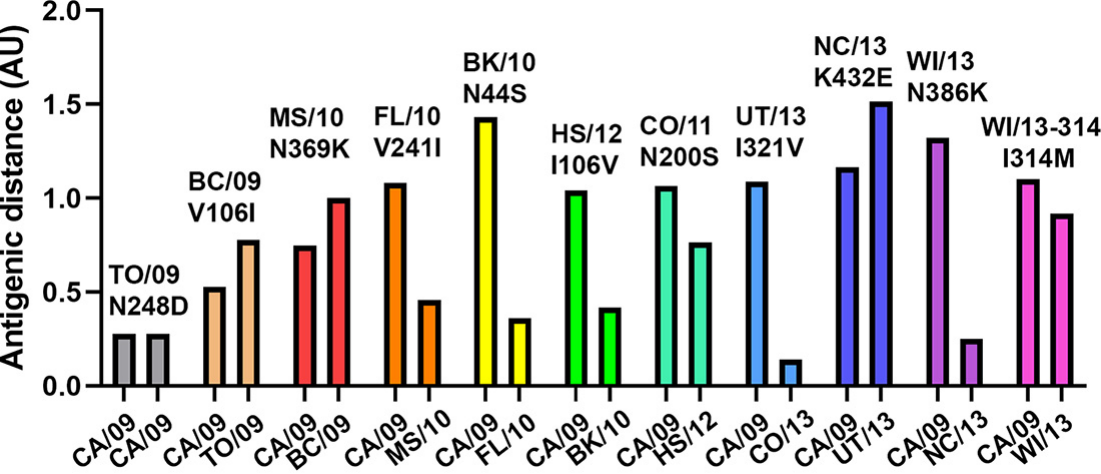
Antigenic distance of NA relative to its precursor NA. For each NA protein (indicated above the bars, including the substitution relative to its precursor), the antigenic distance relative to CA/09 as well as to its precursor NA (indicated below the bars) based on the antigenic map shown in Fig. S5 is graphed. AU, antigenic unit (corresponding to a 2-fold serum dilution).

Substitutions in NA may be selected because of their effect on NA antigenicity. The antigenic properties of the different NAs were tested by performing NA inhibition ELLAs (Table S1) using recombinant soluble NA proteins in combination with a panel of ferret antisera (Table S1 and Fig. S5). Generally, the ferret sera raised against the early viruses (from 2009) displayed the highest titers against the NAs from the early viruses, while the reciprocal effect was observed for the ferret serum raised against the 2015 virus. Thus, the serum raised against A/CA/007/2019 H1N1 displayed inhibition titers of 8.44 (log_2_) against CA/09 and 7.25 (log_2_) against WI/13-314 NA, while the titers of the serum raised against A/NL/148/2015 H1N1 were 7.08 (log_2_) against CA/09 and 8.19 (log_2_) against WI/13-314 NA (Table S1). Based on the results shown in Table S1, an antigenic map was generated (Fig. S6). Distances between proteins and sera in the map correlated well with the respective raw ELLA NI titers (*R*^2^ = 0,8671), indicating that the two-dimensional (2D) map is a good representation of the data and can be used to extrapolate antigenic distances between the NAs. The antigenic map was used to determine the antigenic distance between the different NA proteins relative to CA/09 and to their immediate precursor protein (Fig. 7) according to the phylogenetic analysis (Fig. 1). Of note, antigenic distances between NAs obtained with recombinant NA proteins appeared smaller than those obtained with virus preparations (37). The antigenic distance to CA/09 gradually increased until the BK/10 NA, after which the distance to CA/09 remained relatively constant. When the antigenic distances of the NAs relative to their precursors were analyzed, which result from a single substitution, the largest antigenic change resulted from the K432E substitution introduced in NC13. The N369K substitution in MS/10, which is located in the 2SBS, like K432E, also had a relatively large effect on NA antigenicity.

10.1128/mBio.00287-21.5 FIG S5 Alignment of the N1 proteins of the viruses used to generate the ferret sera is shown together with CA/09 and WI/13-314 NA. Differences from CA/09 are shaded grey or green. The latter color indicates substitutions analyzed in this study. Download FIG S5, TIF file, 0.7 MB. Copyright © 2021 Dai et al. 2021 Dai et al. https://creativecommons.org/licenses/by/4.0/ This content is distributed under the terms of the Creative Commons Attribution 4.0 International license.

10.1128/mBio.00287-21.6 FIG S6 H1N1pdm09 NA antigenic map. Inhibition of NA activity of recombinant NA proteins by the ferret sera was measured by ELLA (Table S1). The antigenic relatedness was calculated (69) using mean titers of 2 or 3 experiments performed in duplicate/triplicate. To determine the rigidity confidence area for each individual antigen (red shapes) and antiserum (shapes with grey outlines), all other antigens and antisera in the map were held fixed, and the target antigen or antiserum was displaced to determine how the error function changed due to the displacement. The periphery of the shapes in the figure indicates an increase of 0.5 in the error function of the optimization. Shading illustrates the rate of error increase for each antigen, from black (no error) to the base color of the antigenic (0.5 error) at the periphery. Antigenic distances between NAs shown in Fig. 7 were determined by measuring distances between NAs on the 2D map. Substitutions in NA relative to the precursor protein (Table 1) are indicated; arrows indicate the evolutionary track according to the phylogenetic tree shown in Fig. 1. Download FIG S6, TIF file, 1.4 MB. Copyright © 2021 Dai et al. 2021 Dai et al. https://creativecommons.org/licenses/by/4.0/ This content is distributed under the terms of the Creative Commons Attribution 4.0 International license.

10.1128/mBio.00287-21.7 TABLE S1 NA inhibition titers. Inhibition of NA activity of recombinant NA proteins by ferret sera raised against different H1N1pdm09 viruses (Fig. S5) was measured by ELLA. Titers were used to generate the antigenic map shown in Fig. S6. Download Table S1, DOCX file, 0.02 MB. Copyright © 2021 Dai et al. 2021 Dai et al. https://creativecommons.org/licenses/by/4.0/ This content is distributed under the terms of the Creative Commons Attribution 4.0 International license.

## DISCUSSION

**FIG 8:**
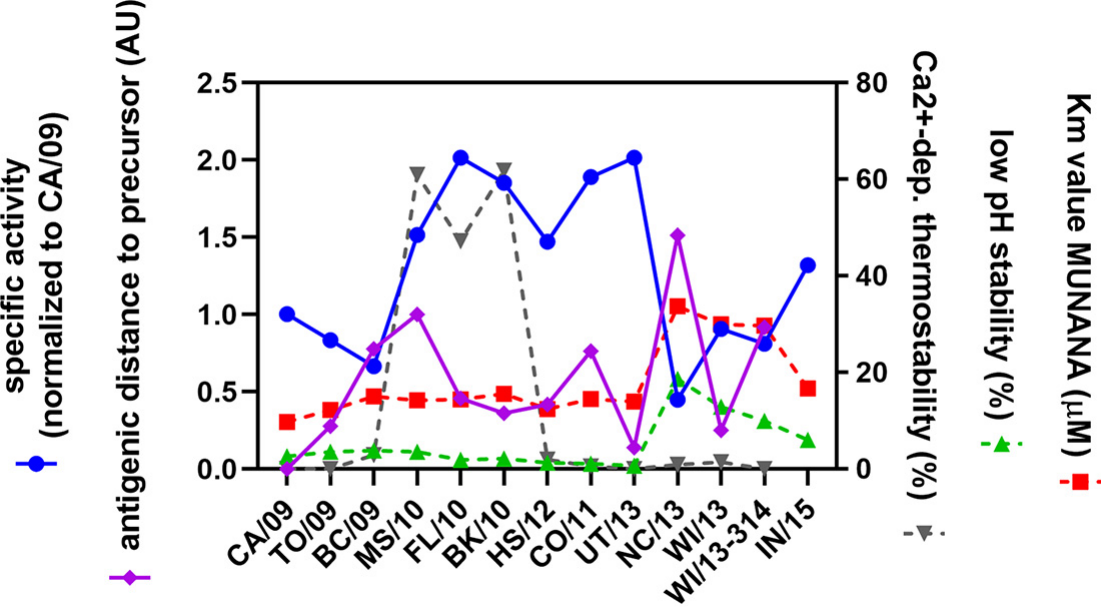
Summary of evolution of N1 phenotypes. The evolution of different NA phenotypic characteristics is summarized. On the left *y* axis, the specific activity as determined using the ELLA (mean of all fetuin-lectin combinations) (Fig. 4) normalized to that of CA/09 and the antigenic distance of each NA relative to its precursor (in AU) (Fig. 7) is represented. On the right *y* axis, the Ca^2+^-dependent thermostability (percent activity relative to unheated controls) (Fig. S2), low-pH stability (percent activity at pH 4.6 relative to pH 6.0) (Fig. 3D), and *K_m_* value (Fig. 3B) are represented.

Relatively little is known, at least compared to HA, regarding to what extent and how changes in NA affect replication and transmission. Adaptation to a novel host sialome and escape from the host immune system are likely driving forces for the selection of substitutions in HA and NA genes upon zoonotic transfer of IAVs. Sixteen amino acid substitutions in NA became fixed in the H1N1pdm09 virus population between 2009 and 2015. The selection of these residues and their being maintained within the virus population are indicative of their biological relevance. The rapid introduction of several substitutions in NA that became fixed within 2 years after introduction of this virus in 2009 is suggestive of adaptation to the human host. These substitutions increased enzymatic activity and affected antibody binding to a modest extent (Fig. 8). NA activity subsequently fluctuated with time within a certain bandwidth rather than being maintained at a distinct optimum. Particularly low NA activity, at least for multivalent substrates, was observed after the substitution K432E in the 2SBS (in NC/13 NA), indicating that low-affinity binding via this site may also enhance NA catalytic activity for a human virus. The K432E substitution also altered antigenicity of NA, which may explain its selection. While the K432E substitution was maintained, enzymatic activity of subsequent NAs increased again by additional substitutions. We note that several substitutions in NA affected more than one NA phenotype, which we propose to be an important factor in the evolutionary trajectory of NA.

None of the substitutions that were conserved in NA involve catalytic or framework residues, in agreement with these residues being extremely conserved in all NA genotypes (15, 16). Of the several substitutions that are located relatively close to the active site (N200S, N248D, V241I, and K432E), K432E had the largest effect on NA catalytic activity. While the specific activity of NA for MUNANA was not affected by this substitution, it increased the *K_m_* value for MUNANA, decreased cleavage of a NeuGc substrate, and resulted in a more active NA at low pH. Residue 432 is located in the 2SBS of NA at a position immediately adjacent to the catalytic site. It interacts via hydrogen bonds with S367, which is an immediate neighbor of the catalytic site residue R368 (Fig. 2). Substitution K432E, which resulted in the formation of two additional hydrogen bonds with water molecules, may therefore indirectly affect the catalytic site (Fig. 2). Substitutions at positions 248 and 106 that were previously reported to increase low-pH activity of NA (19) had only minor effects on low pH activity in our assays. Substitution of residue 106, together with substitution of residue 369, affected Ca^2+^-dependent thermostability of NA, in agreement with findings of a recent study (43).

NAs of most avian IAVs contain a functional 2SBS that, by binding to α2,3-linked sialosides, contributes to catalytic activity on multivalent substrates (12, 13, 22). NA of H1N1pdm09 contains, like all NAs of human IAVs, substitutions of SIA contact residues in the 2SBS. Thus, CA/09 carries an N at position 369 rather than an S, which is found in N1 of avian viruses (12, 21). This N residue was subsequently replaced by K in MS/10 NA, which results in loss of a hydrogen bond with the SIA contact residue S367 (Fig. 2). Somewhat later, K at position 432 was replaced by E in NC/13. N369K increased while K432E decreased cleavage of fetuin-containing α2,3-linked SIAs but not that of monovalent substrates or of transferrin containing only α2,6-linked SIAs. The altered cleavage exclusively of sialosides on fetuin corresponds with the preferred binding of NA via its 2SBS to α2,3-linked SIAs (12, 13, 57), although for this N1 it may be very weak, as we were not able to demonstrate SIA binding via the 2SBS directly. We conclude that low affinity binding of the 2SBS of N1 of H1N1pdm09 to α2,3-linked SIAs can contribute to the catalytic activity of NA. In agreement herewith, previous studies demonstrated binding of H1N1pdm09 NA to SIA via the 2SBS by Brownian dynamics simulation (58) and STD-NMR (26), but with a much lower affinity than for N1 of avian viruses. Of note, the negative effect of K432E on cleavage of multivalent substrates was partly compensated for by N386K, which resulted in the loss of an N-glycosylation consensus sequence. While the loss of an *N*-glycan may explain increased NA activity for multivalent substrates by providing better access to the catalytic site, we did not find proof for the addition of an *N*-glycan to this site. The positive effect of N386K may therefore rather be explained by its localization in a Ca^2+^-binding site (42).

Substitutions in NA were shown to affect NA-driven self-elution from a receptor-coated surface. The reduced self-elution of UT/13 may be explained by the reduced incorporation of NA into virions, while for NC/13, slower self-elution correlates with reduced enzymatic activity against multivalent substrates (Fig. 4). Reduced incorporation of UT/13 NA did not result from this NA being defective in intracellular trafficking and cell surface expression. Substitutions in NA rather resulted in increased cell surface expression levels similarly as observed previously (59). Whether substitutions in NA affect virion self-elution to the same extent when combined with their cognate HAs remains to be determined. Of note, reduced virion self-elution did not appear to affect virus replication much, as virus containing NC/13 replicated with kinetics similar to that of CA/09-containing virus. While well balanced HA and NA activities are important for efficient replication (8–11, 13, 60), the low receptor-binding avidity of CA/09 HA (61) may preclude the necessity for high sialidase activity of virions in our assays, as was observed previously for other IAVs (12, 60, 62).

Immune pressure is known to be a major driving force in IAV evolution. Most substitutions observed in H1N1pdm09 NA are located at the cell surface, several of them in or close to antigenic sites (e.g., positions 248, 369, and 432) (29, 31, 37, 39, 40, 63). Analysis of NA antigenicity using a recombinant protein approach in combination with polyclonal ferret sera revealed a relatively large effect of substitutions in the 2SBS (N369K and K432E). N369K was previously reported to abolish binding of monoclonal antibody HF5 to CA/09 NA, suggesting that it is part of an epitope that may also be targeted by human NA-specific antibodies (39). Antigenic analysis of NA using H1N1pdm09 viruses also suggested an important role for residue 432 in antigenic drift (37), while K432 was shown to be crucial for the binding of several monoclonal antibodies (31, 64, 65). The intertwinement of NA phenotypic properties (Fig. 8), as exemplified by K432E affecting both NA activity and antigenicity, may be an important determining factor in NA evolution. Because of the multiple effects of K432E on NA activity, it may require a receptive background for its selection. Evolution of H1N1pdm09 is furthermore also likely influenced by the importance of a functional HA-NA balance (6). Thus, changes in NA activity and antigenicity, e.g., resulting from K432E, may subsequently drive compensatory substitutions in NA (such as N386K) but also in HA. Conversely, substitutions in HA may also drive the selection of substitutions in NA. Clearly, more research is needed to fully understand the complex functional interplay between HA and NA and the consequences thereof for antigenic evolution.

## MATERIALS AND METHODS

### Phylogenetic analysis.

All full-length and unique NA sequences of A(H1N1)pdm09 viruses in the NCBI database were downloaded (from 2009 to 2015). NA gene trees were constructed by using the PHYLIP neighbor-joining algorithm with the F84 distance matrix. This tree was used as a guide tree to select NA sequences representing all main branches of the tree. The selected NA genes were used to construct a summary tree with topology similar to that of the guide-tree. This unrooted N1 tree is displayed in Fig. 1 with A/California/04/2009 at the base.

### Cell lines.

Human embryonic kidney 293T (HEK293) cells and Madin-Darby canine kidney (MDCK; NBL-2) cells were obtained from the American Type Culture Collection (ATCC) and cultured in Dulbecco’s modified Eagle’s medium (Thermo Fisher Scientific) supplemented with 10% fetal bovine serum (FBS) (Thermo Fisher Scientific), 100 IU/ml penicillin, 100 μg/ml streptomycin, and 0.25 μg/ml amphotericin B (Corning antibiotic-antimycotic solution) at 37°C and 5% CO_2_. Normal human bronchial epithelial (NHBE) cells (Lonza; CC-2540, lot no. 630564) were isolated from a 16-year-old Caucasian female and were differentiated in an air-liquid interface following the manufacturer’s instructions (Lonza; CC-4175) at 37°C and 5% CO_2_.

### NA expression and purification.

Human codon-optimized NA ectodomain (amino acids 42 to 469; N1 numbering)-encoding cDNAs (GenScript, USA) of A/California/04/2009(H1N1) (GenBank accession no. ACP41107.1; referred to as CA/09) and A/Wisconsin/09/2013(H1N1) (GenBank accession no. AGV29183.1; referred to as WI/13) were cloned into a pFRT expression plasmid (Thermo Fisher Scientific) fused to sequences coding for an N-terminal signal sequence derived from *Gaussia* luciferase, a double streptavidin tag for affinity purification (One-STrEP; IBA GmbH), and a Staphylothermus marinus tetrabrachion tetramerization domain, as described previously (12). Mutations of interest (Table 1) were introduced into the NA genes by using the Q5 site-directed mutagenesis kit (New England Biolabs) and confirmed by sequencing. For full-length NA constructs, corresponding NA transmembrane domains were added to the soluble NA-encoding sequences by replacing the terminal signal sequence, streptavidin tag, and tetrabrachion tetramerization domain using conventional cloning. Recombinant soluble NA proteins were expressed by transfection of HEK293T cells with NA-encoding plasmids and purified using Strep-Tactin beads (IBA), as described previously (12). Quantification of the purified proteins was performed by comparative Coomassie gel staining using standard bovine serum albumin (BSA) samples (Sigma-Aldrich) with known concentrations as a reference.

### NA enzymatic assays using soluble NA tetramers.

The activity of serially diluted recombinant soluble NA proteins against monovalent substrates was determined by using a fluorometric assay as described before (34) using either 2′-(4-methylumbelliferyl)-α-d-*N*-acetylneuraminic acid (MUNANA; Sigma-Aldrich) or 2′-(4-methylumbelliferyl)-α-d-*N*-glycolylneuraminic acid (MUNGNA) (66) as the substrate. When indicated, NA samples were incubated at 50°C in the absence or presence of 1 mM EDTA (Sigma-Aldrich) prior to incubation with MUNANA to determine NA thermostability. The MUNANA assay was also performed in 0.1 M citrate reaction buffer (at pH 6.0 or 4.6). NA kinetic analysis was performed to determine the *K_m_* values (substrate binding affinity) of the NA proteins, as described previously (57).

Activity of the recombinant soluble NA proteins toward multivalent glycoprotein substrates was analyzed using a previously described enzyme-linked lectin assay (ELLA) (22, 67). Briefly, fetuin- or transferrin-coated (both from Sigma-Aldrich) 96-well plates were incubated with serial dilutions of recombinant soluble NA proteins in reaction buffer (50 mM Tris/HCl, 4 mM CaCl_2_ [pH 6.0]). After overnight incubation at 37°C, plates were washed and incubated with biotinylated lectins *Erythrina crista-galli* lectin (ECA), Sambucus nigra lectin (SNA), or Maackia amurensis lectin I (MALI) (all from Vector Laboratories). The binding of ECA, SNA, and MALI was detected using horseradish peroxidase (HRP)-conjugated streptavidin (Thermo Fisher Scientific) and tetramethylbenzidine substrate (TMB; BioFX) in an EL-808 enzyme-linked immunosorbent assay (ELISA) reader (Biotek), which read the optical density (OD) at 450 nm. For both the fluorometric assay and the ELLA, the data were fitted by nonlinear regression using Prism 6.05 software (GraphPad). The resulting curves were used to determine the amount of NA protein corresponding to half-maximal activity or lectin binding. The inverse of this amount is a measure for specific activity (activity/amount of protein) and is graphed relative to other NA proteins.

### NA enzymatic assays using cell-associated proteins.

NA activity of cell-associated proteins was also determined using the MUNANA assay, as described previously (59). Briefly, HEK293T cells transfected with equal amounts of full-length-NA-encoding expression plasmids were treated with trypsin-EDTA for 2 min at 72 h posttransfection, followed by the addition of 10% fetal calf serum to neutralize the trypsin. Subsequently, cells were collected by centrifugation for 5 min at 1,500 rpm followed by three washes with Dulbecco's phosphate-buffered saline (PBS). The MUNANA assay was performed in PBS containing 1 mM Ca^2+^ to determine the activity of NA at the cell surface. To determine the total cell NA activity, 0.1% Triton X-100 was added to the reaction buffer.

### Antigenic analysis of NA.

Inhibition of NA activity by the ferret sera was measured by ELLA as described previously, with modifications (22, 68). Ferret sera were raised either against a classical swine H1N1 strain (A/NL/386/86), H1N1pdm09 vaccine strain (A/California/007/09), or several other H1N1pdm09 viruses isolated in the Netherlands (Table S1). Serial dilutions of ferret sera were mixed with purified NA protein diluted in reaction buffer (50 mM Tris/HCl, 4 mM CaCl_2_ [pH 6.0]). The mixture was transferred to 96-well plates coated with 2.5 μg/ml fetuin (Sigma-Aldrich) and incubated at 37°C for 2 h. After extensive washing, the plates were incubated with biotinylated ECA (1.25 μg/ml; Vector Laboratories) at room temperature (RT) for 1 h. The amount of ECA binding was determined as described above. The anti-NA ELLA titers of the sera correspond to the dilutions at which 50% of maximal NA enzyme activity was achieved, as determined by nonlinear regression analysis (GraphPad Prism 6.05). The antigenic relatedness was calculated (69) using mean titers from 2 or 3 experiments performed in duplicate/triplicate. Calculated distances between proteins and sera correlated well with distances on the 2D map (*R*^2^ = 0.8671), allowing us to determine antigenic distances between NA proteins by measuring distances on the 2D map.

### Plasmid-based viral rescue and propagation.

Specific nucleotide substitutions in the A/California/04/2009 NA coding sequence were added to match the sequence of the NA of A/Mississippi/04/2010 (MS/10), A/North Carolina/07/2013 (NC/13), A/Utah/06/2013 (UT/13), and A/Indiana/08/2015 (IN/15) strains (Table 1). This was achieved using the QuikChange mutagenesis kit (Agilent) or by generating specific synthesized oligonucleotides using the GeneArt Strings DNA fragment platform (Thermo Fisher Scientific, Waltham, MA) and subcloning into pDZ vectors. Standard reverse genetics were used to rescue each individual virus in a A/California/04/2009 (Cal09) backbone, as previously described (70). Lipofectamine 2000 (Thermo Fisher Scientific, Waltham, MA) was used to transfect a coculture of HEK293T and MDCK cells with seven ambisense pDZ plasmids, each one encoding a segment of the virus, and using the specific pDZ vector containing either the Cal09 NA or the modified NA cDNAs. Transfected cells were incubated for 72 h at 37°C in a 5% CO_2_ incubator. Cell culture supernatants containing influenza virions were collected. Final stocks of each recombinant virus were achieved after plaque purification and infecting fresh MDCK cells (80% confluence) with a 1:100 dilution of the purified plaque and incubated at 37°C for 48 h in Eagle minimum essential medium (MEM) (Thermo Fisher Scientific) supplemented with 1 μg/ml of TPCK (*N*-tosyl-l-phenylalanine chloromethyl ketone)-trypsin (Sigma-Aldrich). Viral titers were determined by plaque assay and by hemagglutination assays using a suspension of 5% turkey red blood cells in PBS. Absence of defective interfering particles was confirmed by comparing hemagglutination activity versus viral titers, and complete genome sequencing was obtained using the Illumina MiSeq platform to confirm nucleotide substitutions and absence of unexpected changes in the sequence.

### IAV infections.

MDCK cells were inoculated with virus preparations in PBS supplemented with 0.3% BSA (Gemini) in triplicates. After 1 h of incubation at room temperature, cells were washed with DMEM supplemented with 10% FBS to remove the unbound virus. Cells were incubated in MEM supplemented with 0.3% BSA and 1 μg/ml (MDCK cells) of TPCK-trypsin at 37°C in a 5% CO_2_ incubator. When indicated, cell culture supernatants were sampled at different time points to assess viral replication by standard plaque assay technique using MDCK cells. NHBE cells were incubated with the viral inoculum in PBS on the apical surface. After 1 h of incubation at 37°C in 5% CO_2_, the viral inoculum was removed, and the apical surface was washed twice with PBS to remove unbound virus. Cells were incubated without the presence of TPCK-trypsin at 37°C in 5% CO_2_. When indicated, cell culture supernatants were sampled at different time points to assess viral replication by standard plaque assay technique using MDCK cells.

### Analysis of NA activity using BLI.

Biolayer interferometry (BLI) assays were performed using Octet RED348 as described previously (13, 56). All the experiments were performed in PBS with calcium and magnesium (Lonza) at 30°C and with shaking of plates at 1,000 rpm. Streptavidin sensors were loaded to saturation with recombinant soluble lysosome-associated membrane glycoprotein 1 (LAMP1) coexpressed with human α-2,6-sialyltransferase 1 (ST6Gal1) to increase levels of α2,6-sialylated glycans (6′LAMP1) (Du et al., submitted). Receptor-loaded sensors were subsequently moved to wells containing viruses to perform virus binding in the presence of NA inhibitor oseltamivir carboxylate (OC; kindly provided by Roche). Amounts of virus stocks used were adjusted so that similar binding levels were obtained and thus that similar numbers of virus particles were captured on the sensors. After 3 short (5-s) washes in PBS, NA-driven dissociation was monitored in the absence of OC. Three washes in PBS are sufficient to remove OC to such an extent that efficient virion self-elution can be observed (13, 56) in agreement with the fast dissociation of OC from NA (71).

### Western blot analysis of virions.

Virion-containing cell culture media were cleared by low-speed centrifugation (258 × *g* for 10 min), after which virions were pelleted through a 20% sucrose cushion in PBS by ultracentrifugation (29,000 rpm for 3 h). The pellets were taken up in PBS and subjected to gel electrophoresis followed by Western blotting analysis using specific antibodies against NA (7D3 [41]; kindly provided by Xavier Saelens) or nucleoprotein (NP) (HB65; kindly provided by Ben Peeters). The antibody against NA recognizes a conserved epitope in NA that is identical for all the NA proteins studied.

### Hemagglutination assay with NA membrane vesicles.

HEK293T cells were transfected with expression plasmids encoding full-length N1 proteins. At 72 h posttransfection, cells were vesiculated as described previously (12) and vesicle preparations were purified using Capto Core 700 beads (GE Healthcare Life Sciences) according to the manufacturer's instructions and as detailed previously (12, 72) to remove proteins smaller than 700 kDa. NA activity in the purified vesicle preparations was determined using the MUNANA assay described above. Hemagglutination assays were performed with membrane vesicles containing similar amounts of NA activity of either the indicated H1N1pdm09 NAs or NAs (N1_Hunan_ and N1_432E_) from H5N1 viruses (Du et al., submitted) as described previously (12). Twofold serial dilutions of the vesicles were incubated with equal volumes of 0.5% human erythrocytes (Sanquin) at 4°C for 2 h in the presence of OC.

### Statistical analysis.

The mean values from 2 to 6 experiments performed in duplicate/triplicate are graphed. All statistical analyses were performed by one-way analysis of variance (ANOVA) using Tukey's multiple-comparison test (GraphPad Prism 6.05).
