# Supplementary figures and images for: Analysis of the Evolution of Pandemic Influenza A(H1N1) Virus Neuraminidase Reveals Entanglement of Different Phenotypic Characteristics
Source: mBio. 2021 May 11;12(3):e00287-21. doi: 10.1128/mBio.00287-21 (PMC8262965; doi:10.1128/mBio.00287-21)

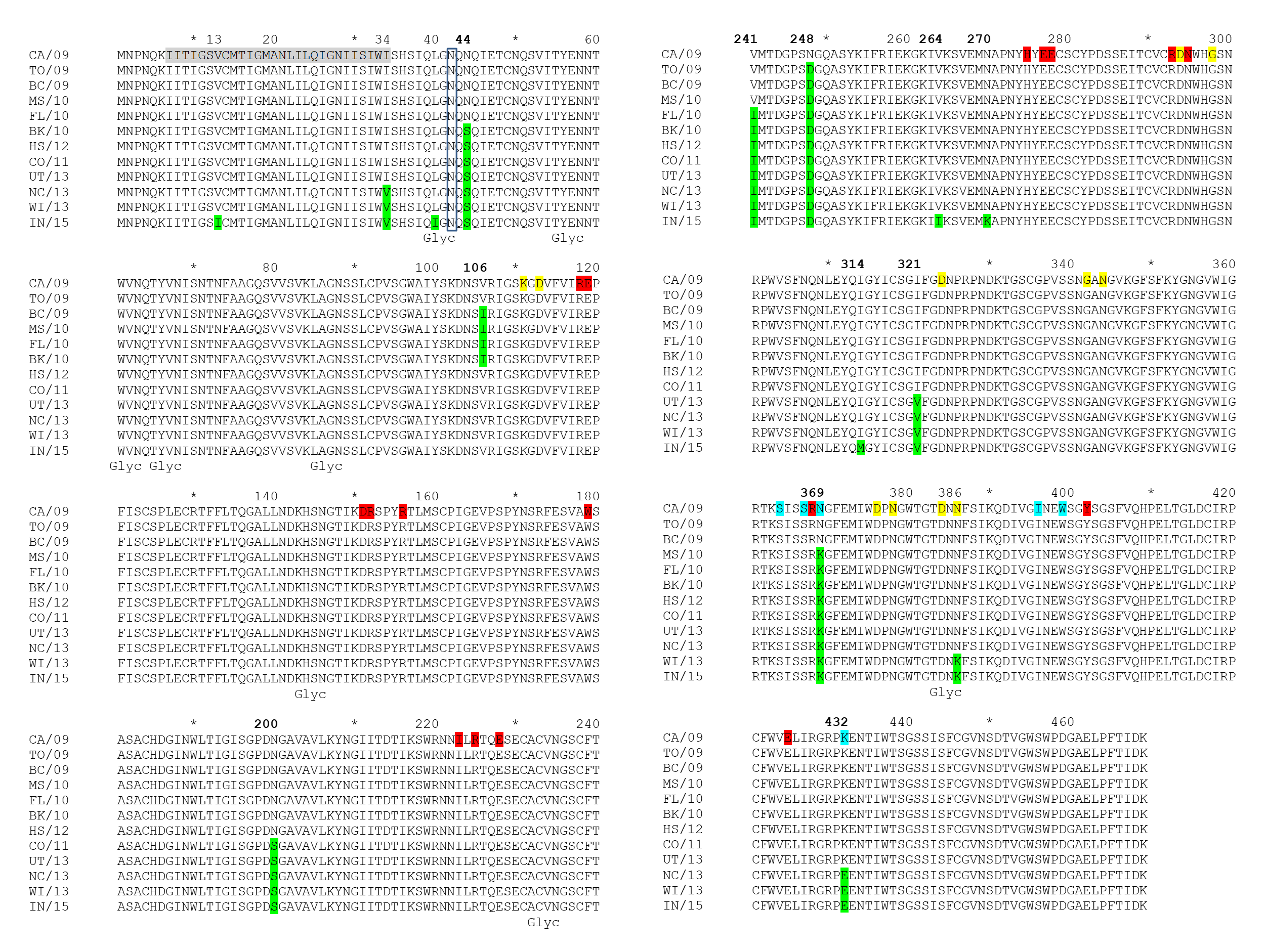

Supplement: FIG S1 [file mbio.00287-21-sf001.tif]

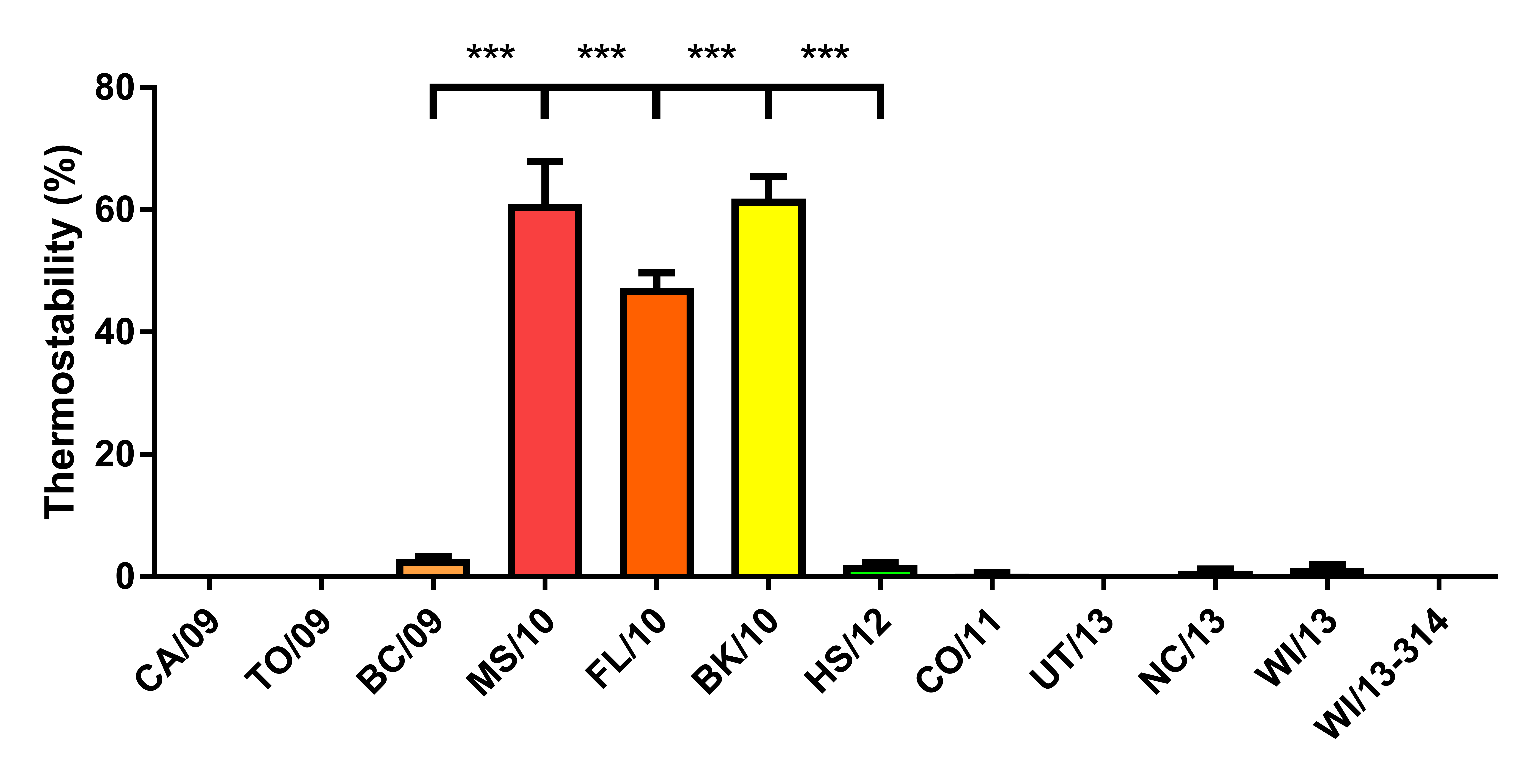

Supplement: FIG S2 [file mbio.00287-21-sf002.tif]

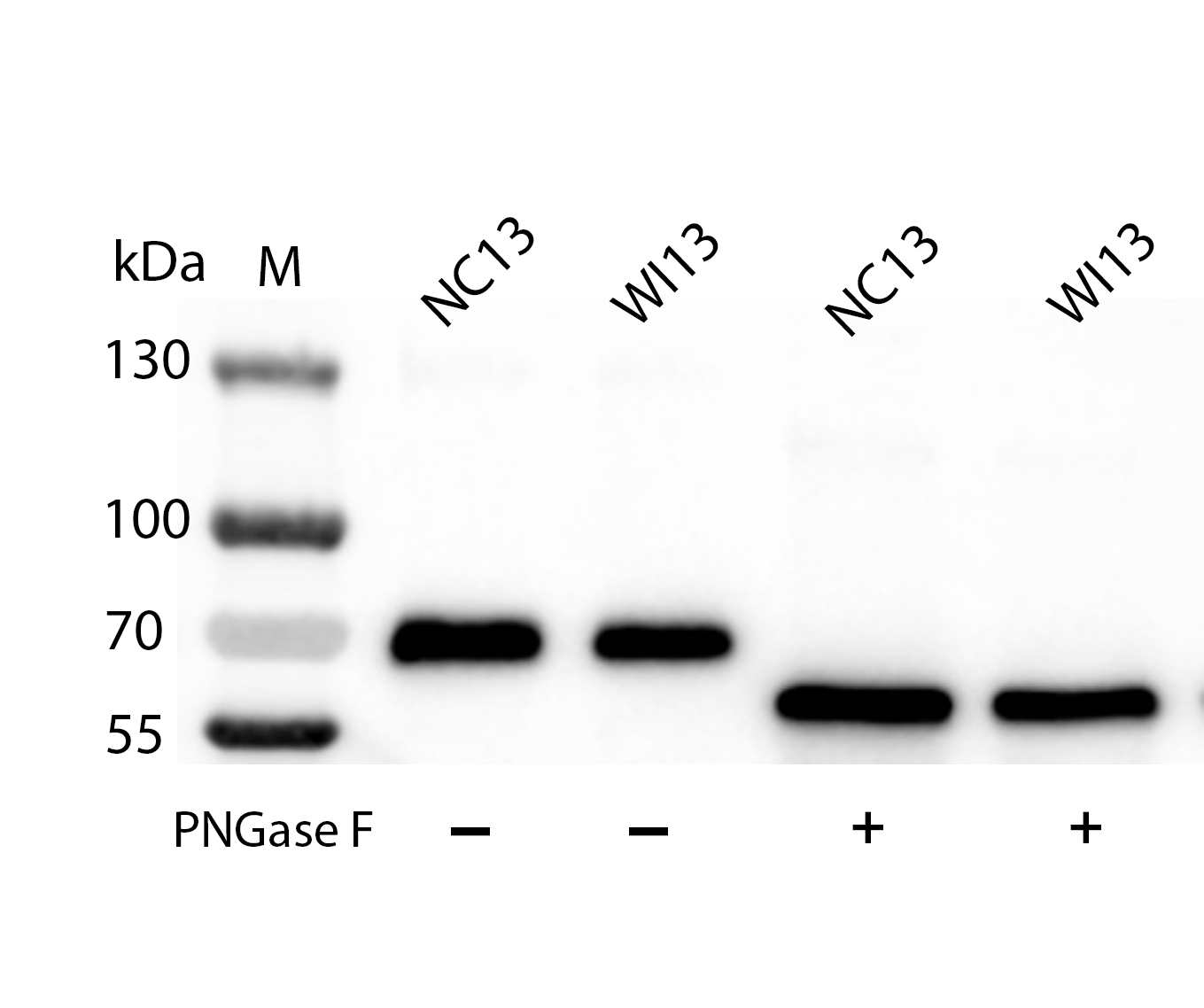

Supplement: FIG S3 [file mbio.00287-21-sf003.tif]

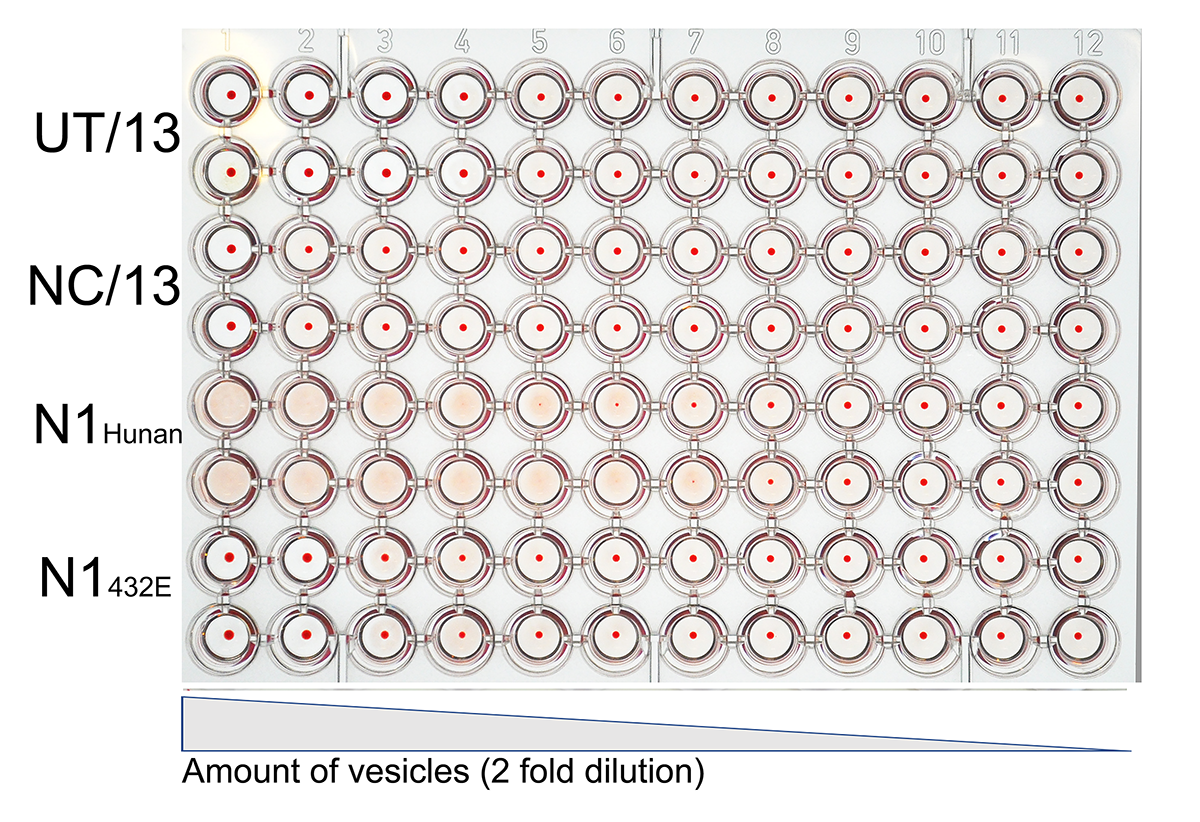

Supplement: FIG S4 [file mbio.00287-21-sf004.tif]

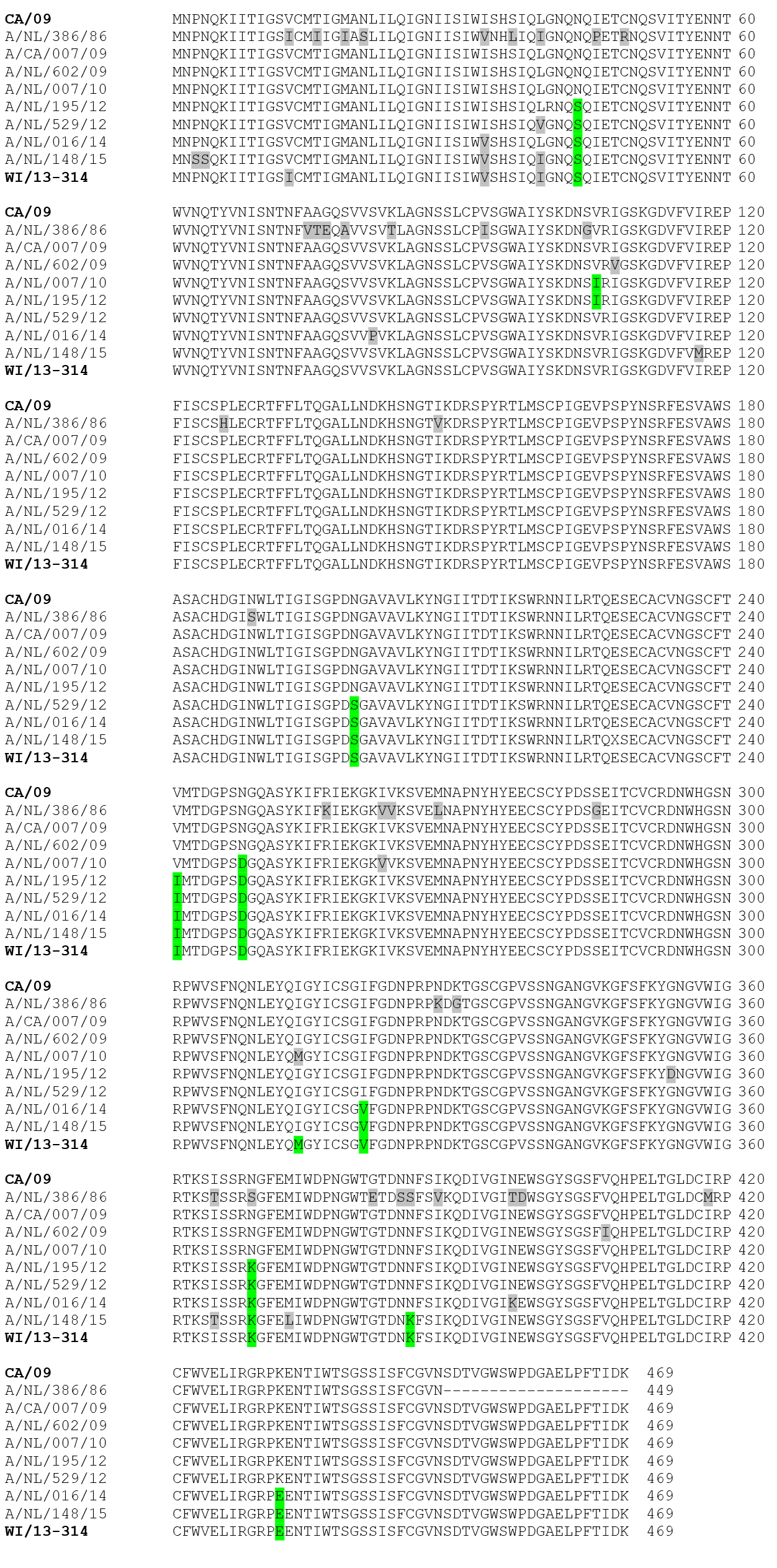

Supplement: FIG S5 [file mbio.00287-21-sf005.tif]

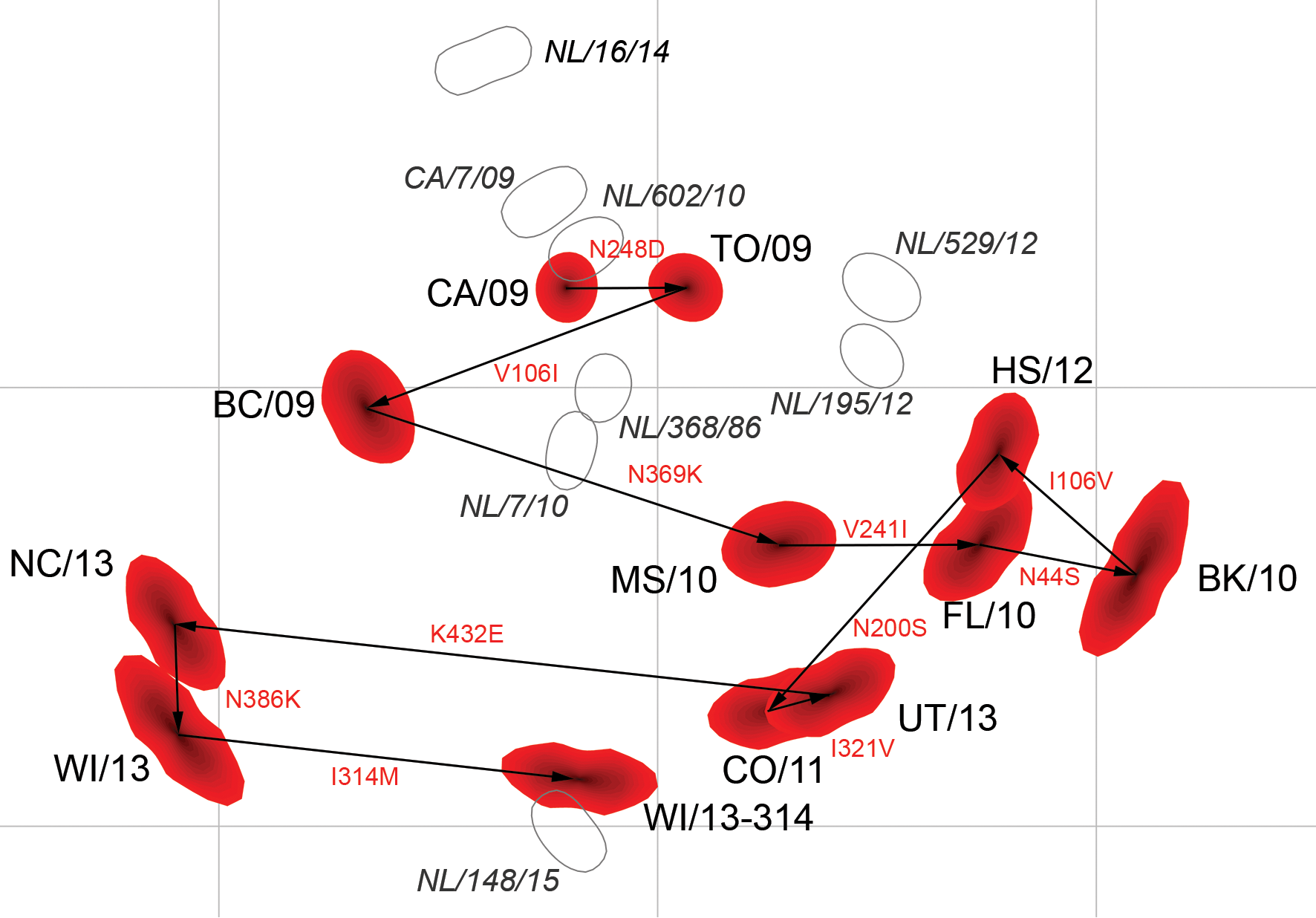

Supplement: FIG S6 [file mbio.00287-21-sf006.tif]
