# Supplementary material for: Analysis of the Evolution of Pandemic Influenza A(H1N1) Virus Neuraminidase Reveals Entanglement of Different Phenotypic Characteristics
Source: mBio. 2021 May 11;12(3):e00287-21. doi: 10.1128/mBio.00287-21 (PMC8262965; doi:10.1128/mBio.00287-21)
Supplement: TABLE S1 [file mbio.00287-21-st001.docx]

**Table S1. NA inhibition titers**

|  |  | Ferret serum raised against | | | | | | | |
| --- | --- | --- | --- | --- | --- | --- | --- | --- | --- |
|  |  | A/NL/386/86 | A/CA/007/09 | A/NL/602/09 | A/NL/007/10 | A/NL/195/12 | A/NL/529/12 | A/NL/016/14 | A/NL/148/15 |
| Antigen and consecutive mutations^a^ |  |  |  |  | V106I  N248D | N44S  V241I  N248D  N369K | I106V  N200S | I321V  K432E | N386K |
| CA/09 | Mean^b^ | 7.78 | **8.44** | **8.00** | 8.00 | 8.90 | 8.69 | 8.92 | 7.08 |
|  | SD | 0.71 | **1.15** | **0.69** | 0.80 | 0.58 | 1.30 | 0.95 | 0.30 |
| TO/09 | Mean | 7.55 | 8.12 | 7.97 | 7.64 | 9.19 | 9.66 | 8.81 | 7.22 |
| N248D | SD | 0.28 | 0.10 | 0.12 | 0.31 | 0.62 | 0.71 | 0.43 | 0.16 |
| BC/09 | Mean | 7.27 | 7.85 | 7.12 | **7.69** | 8.18 | 8.58 | 7.90 | 6.78 |
| V106I | SD | 0.28 | 0.68 | 0.28 | **0.62** | 0.64 | 0.95 | 0.84 | 0.08 |
| MS/10 | Mean | 7.76 | 7.35 | 7.13 | 7.41 | 8.79 | 8.86 | 7.38 | 7.14 |
| N369K | SD | 0.20 | 0.48 | 0.12 | 0.45 | 0.82 | 0.70 | 0.60 | 0.14 |
| FL/10 | Mean | 7.07 | 7.14 | 7.00 | 7.24 | 8.73 | 8.87 | 7.08 | 6.77 |
| V241I | SD | 0.45 | 0.41 | 0.20 | 0.41 | 0.67 | 0.70 | 0.23 | 0.34 |
| BK/10 | Mean | 6.60 | 6.90 | 6.52 | 6.85 | **8.48** | 8.71 | 6.84 | 6.61 |
| N44S | SD | 0.10 | 0.13 | 0.29 | 0.26 | **0.64** | 0.65 | 0.12 | 0.09 |
| HS/12 | Mean | 6.67 | 7.13 | 6.88 | 7.17 | 9.24 | 9.38 | 7.13 | 6.98 |
| I106V | SD | 0.07 | 0.04 | 0.15 | 0.13 | 0.10 | 0.04 | 0.10 | 0.24 |
| CO/11 | Mean | 7.04 | 7.13 | 6.78 | 7.35 | 8.55 | **8.71** | 7.08 | 7.72 |
| N200S | SD | 0.35 | 0.15 | 0.14 | 0.21 | 0.47 | **0.52** | 0.09 | 0.59 |
| UT/13 | Mean | 6.86 | 7.07 | 6.84 | 7.16 | 8.58 | 8.86 | 7.21 | 7.66 |
| I321V | SD | 0.58 | 0.21 | 0.18 | 0.24 | 0.38 | 0.31 | 0.23 | 0.38 |
| NC/13 | Mean | 7.09 | 7.09 | 7.04 | 7.36 | 7.12 | 7.52 | **7.18** | 7.12 |
| K432E | SD | 0.40 | 0.40 | 0.25 | 0.44 | 0.66 | 0.63 | **0.56** | 0.70 |
| WI/13 | Mean | 6.66 | 6.86 | 6.71 | 6.96 | 7.25 | 7.65 | 7.25 | 7.33 |
| N386K | SD | 0.24 | 0.36 | 0.19 | 0.58 | 0.37 | 0.65 | 0.40 | 0.56 |
| WI/13-314 | Mean | 6.39 | 7.25 | 6.52 | 7.42 | 8.03 | 8.62 | 7.64 | **8.19** |
| I314M | SD | 0.27 | 0.14 | 0.08 | 0.21 | 0.32 | 0.36 | 0.33 | **0.32** |

^a^ Column below indicates recombinant NA proteins used in the NA inhibition enzyme-linked lectin assay (ELLA) and the consecutive substitutions in these proteins. Substitutions shown in the row horizontally are indicated when (some) substitutions in the viruses used to generate the ferret sera (indicated above) correspond with the substitutions in the NA antigens. Except for V106I, substitutions indicated are also present in the subsequent viruses of more recent date.

^b^ Mean titers and standard deviations (SD) (Log2) of 2-3 experiments performed in duplicate/triplicate are shown. The titer to the NA protein that is the closest relative to the virus used to generate the ferret serum (0-2 amino acid differences) is shown in bold.
